# On the Chemistry and Physiological Action of the Salicyl Compounds

**Published:** 1891-08-29

**Authors:** 


### THERAPEUTICS.

### ON THE CHEMISTRY AND PHYSIOLOGICAL ACTION OF THE SALICYL COMPOUNDS.

In view of the very frequent employment of the various salicyl compounds in the treatment of rheumatic arthritis and tonsillitis, it may be interesting to briefly review our present knowledge of their chemical nature and relationships, of the toxic results which may ensue from their administration, and of the form and doses in which they are best given in these diseases.

The substances we have to consider are oleum gaultheriæ, salicin, salol, salicylic acid. Taking salicylic acid first, we find that, chemically, it is ortho-oxybenzoic acid, $C_6H_4.OH.CO_2H$, derived from phenol (carbolic acid), $C_6H_5.OH$, by the substitution of one atom of hydrogen in the benzene ring by the group $CO_2H$. Salol is phenyl salicylate $C_6H_5.OH.CO_2C_6H_5$, derived from salicylic acid by replacement of the atom of hydrogen in the group $CO_2H$ by the radicle phenyl, $C_6H_5$. Oil of wintergreen consists mainly of methyl salicylate, $C_6H_4.OH.CO_2CH_3$, while salicin is a glucoside which, by heating with caustic potash and then treating the mass produced with hydrochloric acid, is converted into glucose and salicylic acid.

For some time the only salicylic acid in the market was produced from salicin and from oil of wintergreen; but Kolbe's discovery, in 1874, showed that salicylic acid can be produced from phenol by first heating this with caustic soda, by which sodium phenol, $C_6H_5.ONa$, is produced, and then passing a current of carbon dioxide over this, thus producing sodium salicylate. By this process, which, as is well known, was patented, large quantities of artificial salicylic acid and its salts have been produced, and employed in medicine very extensively, owing to its cheapness as compared with the salicylic acid produced from salicin and oil of wintergreen. It was discovered, however, by the late John Williams, that this synthetically produced salicylic acid had not exactly the same physical and chemical properties as that prepared from salicin; and from his investigations, which have been completed by Professor Dunstan, it would appear that the salicylic acid which was artificially produced had two impurities, ortho- and meta-creostic acids, $C_6H_3.CH_3.OH.CO_2H$ derived from the cresol $C_6H_4.CH_3.OH$, which existed as an impurity in the phenol, from which the salicylic acid was made. (It will be noted that these cresotic acids bear the same relation to the cresol from which they are derived, as does salicylic acid to the phenol from which it takes origin.) However, from Dunstan's researches, it seems that the impurity which exists in the salicylic acid, now made artificially, is no longer ortho- or meta-cresotic acid, but an isomeride, para-cresotic acid. This is probably partly due to differences in the phenol used, also possibly to the fact that the salicylic acid is now made by passing carbon dioxide over the sodium phenol in the cold, and then heating this to a temperature of 140°C, and not, as formerly, at a temperature of 250°C.

We know from the researches of Professor Charteris, to be referred to later, on the toxic properties of para-cresotic acid, that it is of importance to have this artificial acid quite pure, and Professor Dunstan has shown that it is quite easy to separate out the para-cresotic acid by taking advantage of the greater solubility of its lead salt in dilute alcohol.

Pure salicylic acid, so produced, or derived from salicin, is found to have a melting point of 156·75°C, and if a hot aqueous solution is slowly cooled, to crystallise in large prisms, whilst para-cresotic acid is found to have a melting point of 151° C. If, then, it is found that a specimen of salicylic acid has a melting point lower than 156·75° C., it may be concluded that it is not pure, but contains para-cresotic acid.

After this short sketch of chemistry of these compounds, we may pass on to consider which of these drugs is the best for administration. And first, as to salol. As might be suspected from its chemical constitution (a phenyl salicylate), it is split up in the alimentary canal, by the pancreatic juice, into salicylic acid and phenol (or carbolic acid). The phenol so formed, produces carbolurea, and other symptoms of carbolic acid poisoning, and the administration of salol is so only equivalent to giving 64 parts of salicylic acid mixed with 36 parts of this poisonous phenol. The obvious conclusion is that salol should never be given. And next, as to salicin. This has the disadvantage, certainly in the case of children, of possessing a bitter taste; further, in the body it is split up, and sodium salicylate is formed—and it is calculated that 20 grains of salicin produces 15 grains of salicylate of sodium. Salicin then, is only equivalent to a smaller dose of salicylate of sodium, and if the latter is pure, has not the slightest advantage over this drug.

Salicylic acid itself is not very suitable for internal administration on account of its slight solubility in water, though this may be increased by the addition of neutral salts. The conclusion, then, is that by far the most suitable drug to administer is pure salicylate of sodium, prepared either from salicin, or synthetically with subsequent purification in the way above described.

If large quantities of any one of these salicyl compounds be given, toxic effects may arise. These may just shortly be noted in view of what follows; they are deafness, tinnitus, vomiting, delirium, enfeeblement of the heart's action, hæmorrhages, *e.g.*, epistaxis, bleeding from the gums, and hæmaturea. With regard to these one or two points are of practical importance; the first is that hæmorrhages never occur until after the first mentioned toxic symptoms. As soon, then, as these happen it may be concluded that the patient is well under the influence of the drug, and the dose should be reduced. On account of the occasional enfeeblement of the heart's action it is always advisable to give some stimulant along with the drug; for instance, four grains of ammonium carbonate with every twenty grains of salicylate of sodium is a good form of combination. The delirium which occasionally happens must be carefully distinguished from that which may be a symptom of hyperpyrexia; its occurrence should immediately lead to taking the patient's temperature.

The discovery of these various toxic effects of salicylates, and of the chemical impurities which exist in synthetically produced salicylic acid, lead at first to the supposition that the first were results of the second. It is, however, quite certain that all the above toxic effects may occur if only salicin, or salicylic acid prepared from this, be given. Also the researches of Professor Charteris, although incomplete, show that the impurity, para-cresotic acid, which exists in the artificially prepared drug, has as its sole toxic effect a paralysis beginning in the hind legs, and gradually spreading over the body. This, it is obvious, is not seen as a result of the administration of artificial salicylates to man. However, it is best to use only the purified salicylates in future, inasmuch as the para-cresotic acid may possibly be accountable for at least an aggravation of these well-known toxic actions.

The rules, then, for the administration of salicyl compounds in rheumatic arthritis may, perhaps, be formulated as follows: (1) Use only purified salicylate of sodium; (2) give it in doses of 15 20 grains, with 2-4 grains of carbonate of ammonia, every two hours until the temperature is normal or toxic effects ensue, and then less often, or a smaller dose at the same intervals; (3) if delirium occurs, exclude hyperpyrexia as its cause; (4) continue the salicylate for at least a fortnight after the subsidence of the pyrexia, for the occurrence of relapses is probably dependent, to a very great extent, on the cessation of the administration of the drug.

